# A Survey on Artificial Intelligence in Posture Recognition

**DOI:** 10.32604/cmes.2023.027676

**Published:** 2023-04-23

**Authors:** Xiaoyan Jiang, Zuojin Hu, Shuihua Wang, Yudong Zhang

**Affiliations:** 1 School of Mathematics and Information Science, Nanjing Normal University of Special Education, Nanjing, 210038, China; 2 School of Computing and Mathematical Sciences, University of Leicester, Leicester, LE1 7RH, UK

**Keywords:** Posture recognition, artificial intelligence, machine learning, deep neural network, deep learning, transfer learning, feature extraction, classification

## Abstract

Over the years, the continuous development of new technology has promoted research in the field of posture recognition and also made the application field of posture recognition have been greatly expanded. The purpose of this paper is to introduce the latest methods of posture recognition and review the various techniques and algorithms of posture recognition in recent years, such as scale-invariant feature transform, histogram of oriented gradients, support vector machine (SVM), Gaussian mixture model, dynamic time warping, hidden Markov model (HMM), lightweight network, convolutional neural network (CNN). We also investigate improved methods of CNN, such as stacked hourglass networks, multi-stage pose estimation networks, convolutional pose machines, and high-resolution nets. The general process and datasets of posture recognition are analyzed and summarized, and several improved CNN methods and three main recognition techniques are compared. In addition, the applications of advanced neural networks in posture recognition, such as transfer learning, ensemble learning, graph neural networks, and explainable deep neural networks, are introduced. It was found that CNN has achieved great success in posture recognition and is favored by researchers. Still, a more in-depth research is needed in feature extraction, information fusion, and other aspects. Among classification methods, HMM and SVM are the most widely used, and lightweight network gradually attracts the attention of researchers. In addition, due to the lack of 3D benchmark data sets, data generation is a critical research direction.

## 1 Introduction

**Figure 1:**
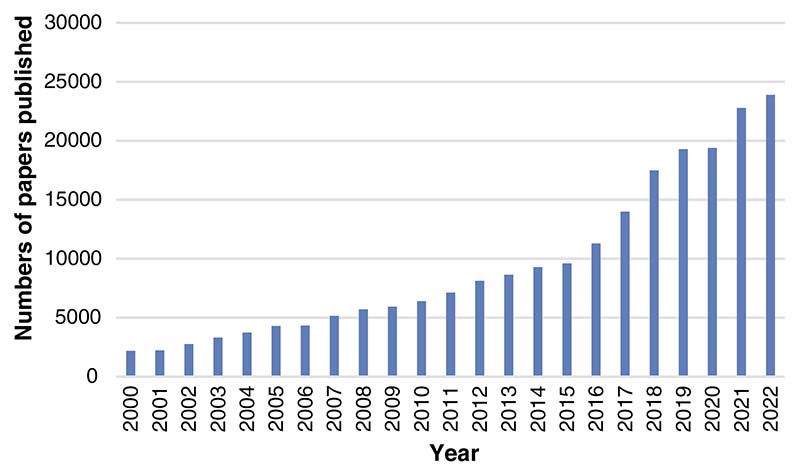
The number of posture recognition papers published (2000–2022)

In recent years, posture recognition has been a research hotspot in computer vision and artificial intelligence (AI) [1], which analyzes the original information of the target object captured by a sensor device or camera through a series of algorithms to obtain the posture. Human body posture recognition has broad market prospects in many application fields, such as behavior recognition, gait analysis, games, animation, augmented reality, rehabilitation testing, sports science, etc. [2]. AI-based posture recognition has also attracted more and more attention from researchers. We retrieved literature on AI-based posture recognition every year from 2000 to 2022, and the number of them showed an increasing trend, as shown in Fig. 1.

Although human posture recognition has become the leading research direction in the field of posture recognition, there are also many studies on animal posture recognition, such as birds [3], pigs [4,5], and cattle [6]. With the rise of artificial intelligence, more and more scholars are interested in the research of posture recognition.

According to the input image type, we generally divide posture recognition algorithms into two categories: algorithms based on RGB images and algorithms based on depth images. The RGB image-based recognition algorithm utilizes the contour features of the human body. For example, the edge of the human body can be described through the histogram of oriented gradients (HOG). The depth-based image algorithm mainly uses the image’s gray value to represent the target’s spatial position and contour. The latter is not disturbed by light, color, shadow, and clothing, but it has higher requirements for information image acquisition equipment [7,8].

The existing posture recognition methods can be summarized into two methods. One is based on the traditional machine learning method, and the other is based on the deep neural network method. In the posture recognition method based on traditional machine learning, the traditional image segmentation algorithm is introduced to realize the segmentation of an image or action video. Then machine learning methods are used for classification, such as support vector machines (SVM), Gaussian mixture model (GMM), and hidden Markov models (HMM). The disadvantage of this method is that the representation ability of these features is limited, representative semantic information is challenging to extract from complex content, and step-by-step recognition lacks good real-time performance.

In the recognition method based on deep learning, the low level-feature information of the image is combined with the deep neural network to estimate and recognize the posture at a higher level. Compared with traditional machine learning algorithms, target detection networks based on deep neural networks often have stronger adaptability and can achieve higher recognition speed and accuracy [9].

**Figure 2:**
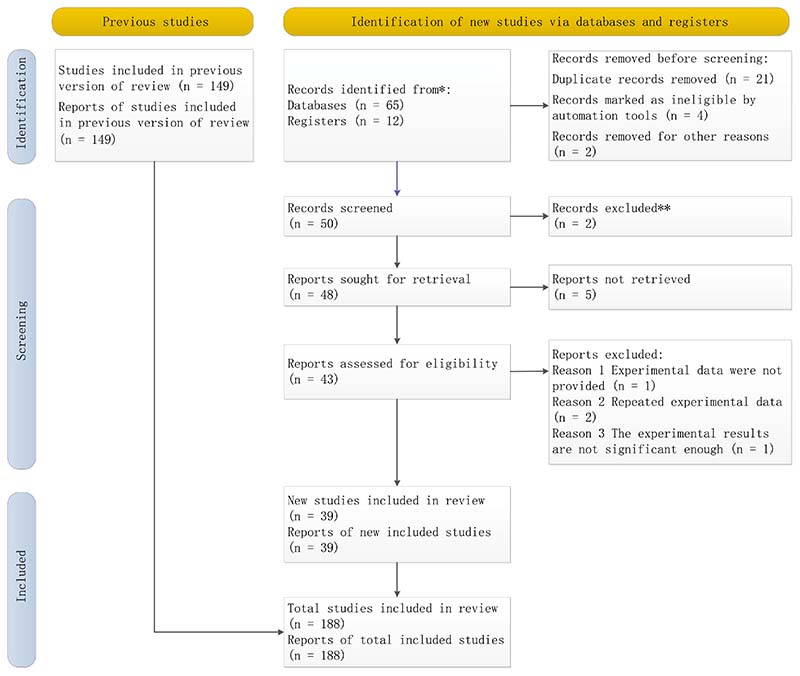
The PRISMA chart of the article selection process for this review

We conducted a systematic review based on the Preferred Reporting Items for Systematic Review and Meta-Analysis (PRISMA). Through Google scholar, Elsevier, and Springer Link, we searched the papers on the application of artificial intelligence to posture recognition. According to the title and content, we eliminated irrelevant and duplicate papers, and finally, the review included 188 papers. The PRISMA chart is shown in Fig. 2.

## 2 Main Recognition Techniques

### 2.1 Sensor-Based Recognition

The sensor-based posture recognition requires the target to wear a variety of sensors or optical symbols and collect the action information of the target object based on this. The research on sensor-based human posture recognition algorithms started earlier. As early as the 1950s, some people used gravity sensors to recognize human posture [10]. In daily human posture recognition research, sensors have been used to distinguish standing, walking, running, sitting, and other stable human posture [11,12].

The common classification methods of posture recognition sensors are as follows. According to the position of the sensor, it can be divided into lower limbs, waist, arm, neck, wrist, etc. Sensors can also be classified according to the number of sensors, which can be divided into single-sensor and multi-sensor. Compared with the method of single-sensor signal processing, the multi-sensor system can obtain more information about the measured target and environment effectively [13,14].

Whether or not the sensor is installed on the user can be divided into wearable and fixed sensors. Wearable devices are a representative example of sensor-based human activity recognition (HAR) [15,16]. The sensor’s type of data output can be divided into array time domain signal, image matrix data, vector data, or strap-down matrix data. Common wearable sensors include inertial sensors (such as accelerometers and gyroscopes), physiological sensors (such as EEG, ECG, GSR, EMG), pressure sensors (such as FSR, bending sensors, barometric pressure sensors, textile-based capacitive pressure sensors), vision wearable sensors (such as WVS), flexible sensors [17].

To avoid physical discomfort and system instability caused by workers on construction sites wearing invasive sensors or attaching multiple sensors to the body, Antwi-Afari et al. [18] utilized the network based on deep learning as well as wearable insole sensor data to automatically identify and classify various postures presented by workers during construction. Hong et al. [19] designed a system using multi-sensors and a collaborative AI-IoT-based approach and proposed multi-pose recognition (MPR) and cascade-adaboosting-cart (CACT) posture recognition algorithms to further improve the effect of human posture recognition.

Fan et al. [20] proposed a squeezed convolutional gated attention (SCGA) model to recognize basketball shooting postures fused by various sensors. Sardar et al. [21] proposed a mobile sensor-based human physical activity recognition platform for COVID-19-related physical activity recognition, such as hand washing, hand disinfection, nose-eye contact, and handshake, as well as contact tracing, to minimize the spread of COVID-19.

### 2.2 Vision-Based Recognition

The vision-based method extracts the information of the key node and skeleton by analyzing the position of each joint point of the target object in the image data. In vision-based methods, cameras are usually used to obtain images or videos that require posture recognition and can be used in a non-contact environment. Therefore, this method does not affect the comfort of motion and has low acquisition costs.

Obtaining human skeleton keypoints from two-dimensional (2D) images or depth images through posture estimation is the basis of vision-based posture recognition. There are inherent limitations when 2D images are used to model three-dimensional (3D) postures, so RGB-D-based methods are ineffective in practical applications. In addition to RGB images and depth maps, skeletons have become a widely used data modality for posture recognition, where skeleton data are used to construct high-level features that characterize 3D configurations of postures [22].

**Figure 3:**
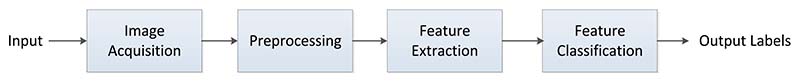
Procedure of vision-based posture recognition

The general process of vision-based posture recognition includes the following: image data acquisition, preprocessing, feature extraction, and feature classification, as shown in Fig. 3.

Currently, video-based methods mainly use deep neural networks to learn relevant features from video images for posture recognition directly. For example, WMS Abedi et al. [23] used convolutional neural networks to identify and classify different categories of human poses (such as sitting, lying, and standing) in the available frames. Tome et al. [24] fused the probabilistic information of 3D human posture with the multi-stage CNN architecture to achieve 3D posture estimation of the original images. Fang et al. [25] designed a visual teleoperation framework based on a deep neural network structure and posture mapping method. They applied a multi-level network structure to increase the flexibility of visual teleoperation network training and use. Kumar et al. [26] used the integration of six independent deep neural architectures based on genetic algorithms to improve the driver’s performance on the distraction classification problem to assist the existing driver-to-pose recognition technology. Mehrizi et al. [27] proposed a computer vision-based label-free motion capture method that combines the discriminative method of posture estimation with morphological constraints to improve the accuracy and robustness of posture estimation.

### 2.3 RF-Based Recognition

In some specific posture recognition situations, the target object cannot wear the sensing device, and radio frequency (RF)-based technology can solve this problem. Due to their non-contact nature, various radio frequency-based technologies are finding applications in human activity recognition. Yao et al. [28] used radio frequency identification (RFID) technology to build a posture recognition system to identify the posture of the elderly, who do not need to wear equipment at this time. Yao et al. [29] used RFID and machine learning algorithms to decipher signal fluctuations to identify activities. Liu et al. [30] proposed a sleep monitoring system based on passive RFID tags, which combined hierarchical recognition, image processing, and polynomial fitting to identify body posture through changes caused by backscattered signals from tags.

Radio frequency signals are extremely sensitive to environmental changes, and changes caused by human movements or activities can be easily captured. Radio frequency signals are absorbed, reflected, and scattered by the body, which will cause changes in the signals. Human activities will cause different changes in the radio frequency signal so that human activities can be identified by analyzing the changes in the signals. The most typically used radio frequency technologies are radar, WiFi, and RFID [31,32].

## 3 Traditional Machine Learning-Based Approach

### 3.1 Preprocessing

Image preprocessing is the basis of posture recognition, which can directly affect the extraction of feature points and the result of posture classification, thus affecting the recognition rate of posture. The main tasks in the preprocessing stage are denoising, human skeleton keypoint detection, scale, gray level normalization, and image segmentation.

The keypoint detection of the human skeleton mainly detects the keypoint information such as human joints and facial features. The output is the skeletal feature of the human body, which is the primary part of posture recognition and behavior analysis, mainly used for segmentation and alignment.

The normalization of scale and gray level should first ensure the effective extraction of key features of the human body and then process the color information and size of the image to reduce the amount of computation.

### 3.2 Feature Extraction

#### 3.2.1 Figure Format Histogram of Oriented Gradients

**Figure 4:**
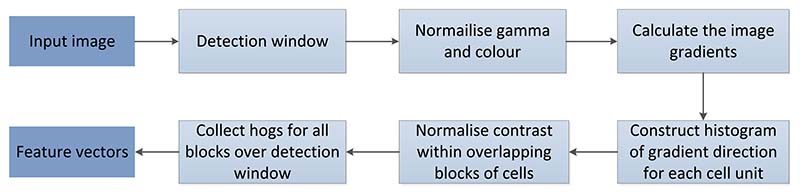
The flowchart of HOG feature extraction

Histogram of oriented gradient (HOG) constitutes features by calculating and statistical histogram of gradient direction in local image regions [33], which describes the entire image region and reflects strong description ability and robustness. HOG classifier is generally combined with the SVM classifier in image recognition, especially in human detection, which has achieved great success. HOG can describe objects’ appearance features and the shape of local gradient distribution [34]. HOG feature extraction steps are as follows in Fig. 4.

(1) The color space of input images is normalized by gamma correction to reduce the influence of light factors and suppress noise interference. Gamma compression is shown in the following formula:
  (1) $$I\left(a,b\right)=I{\left(a,b\right)}^{\gamma }.$$
  Here, the value of gamma has three conditions:
  (i) When gamma is equal to 1, the output value is equal to the input value, and only the original image will be displayed.
  (ii) When gamma is greater than 1, the dynamic range of the low gray value region of the input image becomes smaller, and the contrast of the low gray value region of the image is reduced. In the area of high gray value, as the dynamic range increases, the contrast in the area of high gray value of the image will be correspondingly enhanced. Eventually, the overall gray value of the image will be darkened.
  (iii) When gamma is less than 1, the dynamic range of the low gray value region of the input image becomes larger, and the contrast of the low gray value region of the image is enhanced. In the area of high gray value, if the dynamic range becomes smaller, the contrast in the area of high gray value will decrease accordingly, thus brightening the overall gray level of the image.
(2) The horizontal and vertical gradient values and the gradient direction values of each pixel in the image can be calculated by the following formula:
  (2) $$G_{a}\left(a,b\right)=H\left(a+1,b\right)-H\left(a-1,b\right),$$
  (3) $$G_{b}\left(a,b\right)=H\left(a,b+1\right)-H\left(a,b-1\right),$$
  where *G_a_* (*a*, *b*) represents the horizontal gradient, *G_b_* (*a*, *b*) represents the vertical gradient, and *H* (*a*, *b*) represents the pixel value at pixel point (*a*, *b*).
  The gradient amplitude and orientation at the pixel point are shown in Eqs. (4) and (5), respectively:
  (4) $$G\left(a,b\right)=\sqrt{G_{a}{\left(a,b\right)}^{2}+G_{b}{\left(a,b\right)}^{2}},$$
  (5) $$\alpha \left(a,b\right)=\arctan\frac{G_{b}\left(a,b\right)}{G_{a}\left(a,b\right)}.$$
(3) The gradient orientation histogram is constructed for each cell unit to provide the corresponding code for the local image region. At the same time, the image of the human posture and appearance are kept weak sensitivity.
(4) Every few cell units are formed into large blocks, and the gradient intensity is normalized to realize the compression of illumination, shadow, and edge.
(5) All overlapping blocks in the detection window are collected for HOG features and combined into the final feature vector for classification.

#### 3.2.2 Scale-Invariant Feature Transform

Scale Invariant Feature Transform (SIFT) is an algorithm that maps images to local feature vector sets based on computer vision technology. The essence is to find the keypoints or feature points in different scale-spaces and then calculate the direction of the keypoints [35].

**Figure 5:**
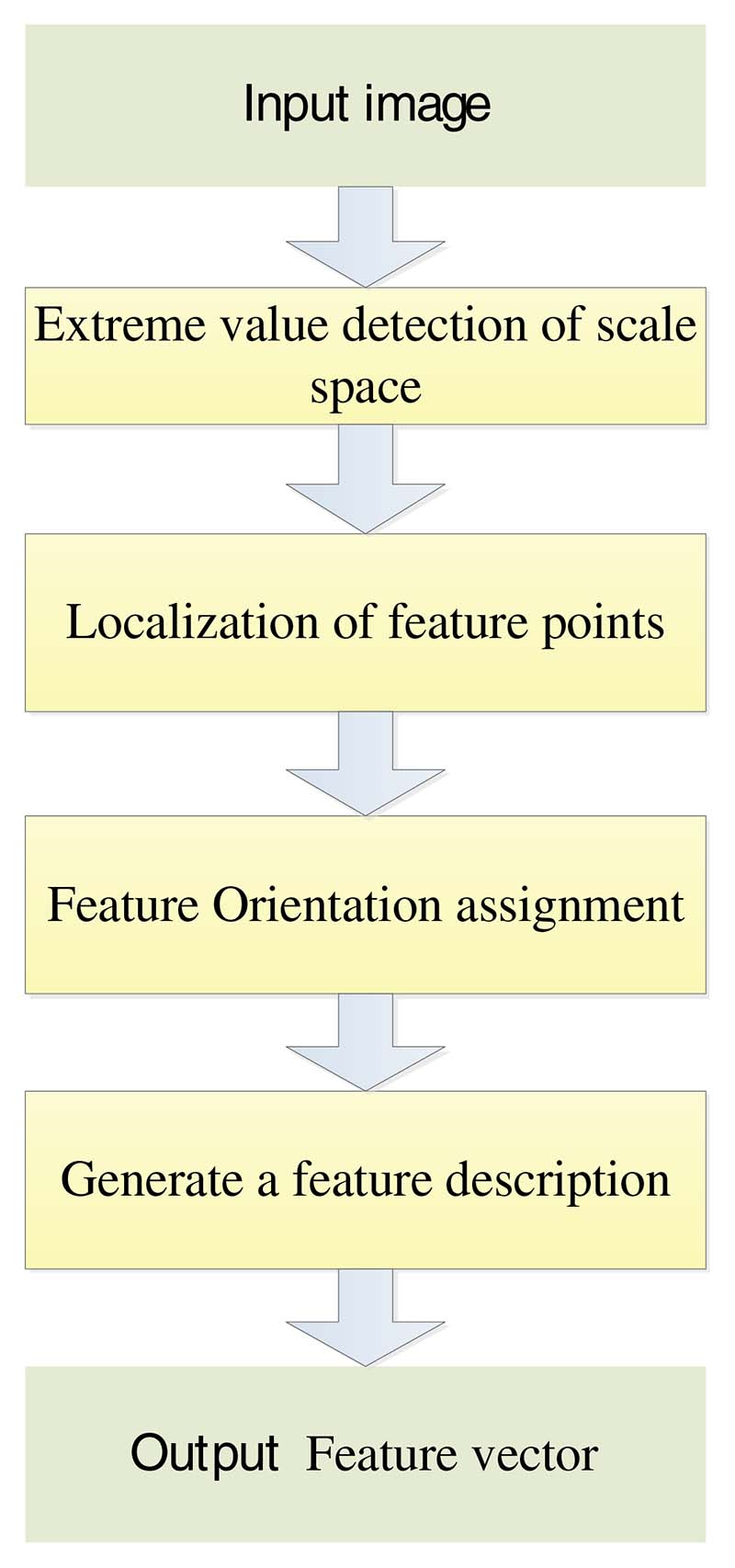
The flowchart of SIFT algorithm

Therefore, SIFT features do not vary with the changes in image rotation, scaling, and brightness and are almost immune to illumination, affine transformation, and noise [36]. Yang et al. [37] used SIFT feature extraction to study writing posture and achieved good results. The main steps of SIFT algorithm are as follows in Fig. 5.

(1) Extreme value detection of scale space
  Images over all scale spaces are searched, and Gaussian differential functions are used to identify potential points of interest that are not affected by scale and selection. This can be done efficiently by using the “scale space” function as follows:
  (6) $$G\left(x,y,\delta \right)=\frac{1}{2\pi \delta^{2}}e^{\frac{x^{2}+y^{2}}{2\delta^{2}}},$$
  (7) S(x,y,δ)=G(x,y,δ)×I(x,y),
  where *G* (*x*, *y*, *δ*) is a Gaussian kernel function, (*x*, *y*) is the space coordinate, *δ* refers to the scale space factor, and *S* (*x*, *y*, *δ*) refers to the Gaussian scale space of the image. The purpose of establishing the scale space is to detect the feature points that exist on different scales. The Gaussian Laplacian operator (LoG) is a good operator for detecting feature points, but its computation is extremely large, so the Gaussian difference (DoG) is usually used to approximate LoG.
  (8) D(x,y,δ)=S(x,y,kδ)−S(x,y,δ).
  Here, *k* is the scaling factor of two adjacent Gaussian scale spaces.
(2) Localization of feature points
  At this stage, we need to remove the points that do not meet the criteria from the list of keypoints. The points that do not meet the requirements are mainly low-contrast feature points and unstable edge response points.
(3) Feature orientation assignment
  One or more directions should be assigned to each keypoint location according to the local gradient direction of the image to achieve rotation invariance. To ensure the invariance of these features, scholars perform all subsequent operations on the orientation, scale, and position of the keypoints. After finding the feature point, the scale of the feature point and its scale image can be obtained:
  (9) $$h\left(x,y\right)=\sqrt{{\left(L\left(x+1,y\right)-L\left(x-1,y\right)\right)}^{2}+{\left(L\left(x,y+1\right)-L\left(x,y-1\right)\right)}^{2}},$$
  (10) $$\theta \left(x,y\right)=\arctan\frac{L\left(x,y+1\right)-L\left(x,y-1\right)}{L\left(x+1,y\right)-L\left(x-1,y\right)}.$$
  Here, *h* (*x*, *y*) and *θ* (*x*, *y*) denote the magnitude and orientation of the gradient at each point *L* (*x*, *y*), respectively. After calculating the gradient direction, the gradient orientation and amplitude of the pixel near the feature point are calculated by the histogram.
  In the histogram, the horizontal axis represents the intersection angle of the gradient orientation, the vertical axis represents the sum of the gradient amplitudes corresponding to the gradient orientation, and the orientation corresponding to the peak value is the primary orientation of the feature points.
(4) Generate a feature description
  After the above operation, the feature point descriptor must be generated, containing the feature points and the pixels around them. In general, the generation of feature descriptors consists of the following steps: (i) To achieve rotation invariance, the main orientation of rotation is corrected.
  (ii) Generate descriptors and form 128-dimensional feature vectors. (iii) Normalize the feature vector length to remove illumination’s influence.

#### 3.2.3 Dynamic Time Warping

In time series analysis, dynamic time warping (DTW) is introduced to compare the similarity or distance between two arrays or time series of different lengths. DTW was initially used in speech recognition and is now widely used in posture recognition [38–41].

Suppose there are two sequences denoted by *P* = [*P*_1_, *P*_2_, …, *P_m_*] and *Q* = [*Q*_1_, *Q*_2_, …, *Q_n_*]. Here, *m* and *n* are the lengths of the two sequences, respectively. When *m* is equal to *n*, the Euclidean distance (formula (11)) can be directly used to calculate the distance *d* between the two sequences.

(11) $$d=\sqrt{\sum_{i=1}^{m}{\left(P_{i}-Q_{i}\right)}^{2}}.$$

When *m* is not equal to *n*, DTW is introduced to regularize the sequence to make it matches. To align the two sequences, construct a matrix grid of *m* × *n*. The elements in the matrix (*a*, *b*) are the distance between *P_i_* and *Q_i_*, that is, the similarity between each point in sequence *P* and each point in sequence *Q*. The smaller the distance, the higher the similarity, and the shortest path from the start to the end. This path is called the “warping path” and is denoted by *W*. The *l*th element of *W* is defined as:

(12) $$W_{l}={\left(a,b\right)}_{l},$$

(13) $$W=w_{1},w_{2},\ldots ,w_{l},\ldots w_{L},\max\left(m,n\right)\leq L<m+n-1.$$

This path needs to satisfy the following constraints [42]:

(1) The order of each sequence part cannot be changed, and the selected path starts at the bottom left corner of the matrix and ends at the top right corner. The boundary conditions must be met, as shown in Eq. (14):
  (14) $$\{\begin{array}{l} w_{1}=\left(1,1\right)\\ w_{L}=\left(m,n\right) \end{array}$$
(2) Ensure that each coordinate in the two sequences appears in the warping path *W*, so a point can only be aligned with its neighboring point.
(3) The points above the warping path *W* must be monotonically progressed over time.

**Figure 6:**
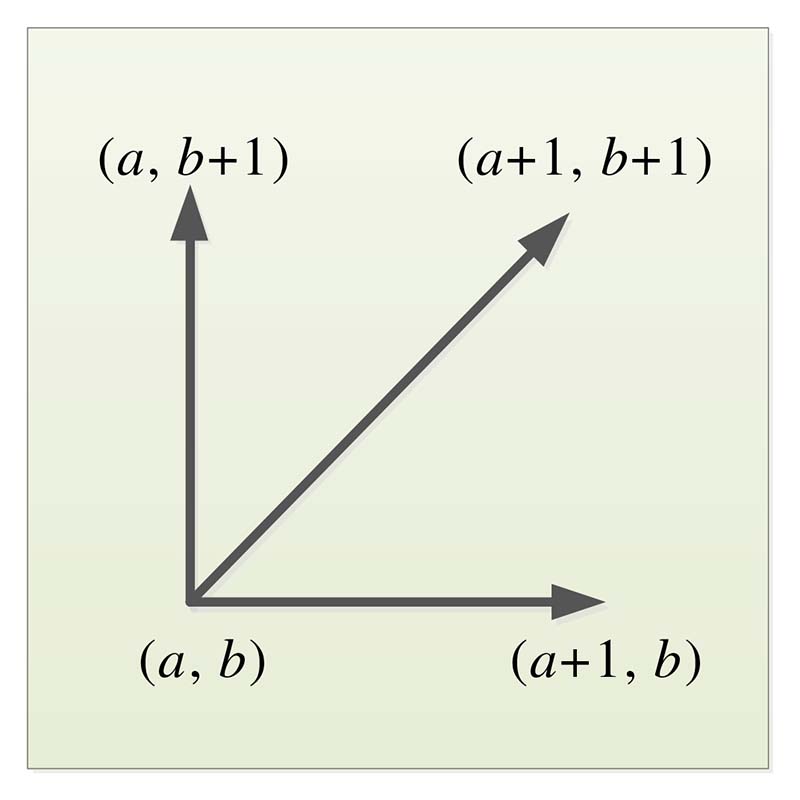
Diagram of the path search direction

Therefore, only three directions to choose the path to each grid point. Assuming the path has already passed through grid point (*a*, *b*), the location of the next grid point to pass through can only be one of three cases: (*a* + 1, b), (*a*, *b* + 1), and (*a* + 1, *b* + 1), as shown in Fig. 6. We can solve the value of DTW according to Eq. (15).

(15) $$DTW\left(P,Q\right)=\min\left\{\frac{\sqrt{\sum_{l=1}^{L}w_{l}}}{L}\right\}.$$

#### 3.2.4 Other Feature Extraction Approaches

In addition to the above two feature extraction methods (HOG [43–46], SIFT [47–49], DTW [50,41,39,40]) for posture recognition, several feature extraction methods are widely used in posture recognition, such as Hu moment invariant (HMI) [51,52], Fourier descriptors (FD) [53,54], nonpara-metric weighted feature extraction (NWFE) [55,56], gray-level co-occurrence matrix (GLCM) [57,58].

### 3.3 Feature Reduction

After feature extraction is completed, feature dimension reduction is needed when the dimension is too high to improve the speed and efficiency of calculation and decision-making. Principal component analysis (PCA) [59] and linear discriminant analysis (LDA) [60] are the most commonly used dimensionality reduction methods.

PCA aims to try to recombine the numerous original indicators with certain correlations into a new set of unrelated comprehensive indicators and then replace the original ones [61]. It is an unsupervised dimensionality reduction algorithm. LDA is a supervised linear dimensionality reduction algorithm. Unlike PCA, LDA maintains data information and makes dimensionality reduction data as easy to distinguish as possible [62].

### 3.4 Classification

#### 3.4.1 SVM

Corinna Cortes et al. first proposed the support vector machine (SVM) to find the optimal solution from two types of different sample data [63]. There may be multiple partition hyperplanes for the sample space to separate the two training samples. SVM is used to find the best hyperplane to separate the training samples.

**Figure 7:**
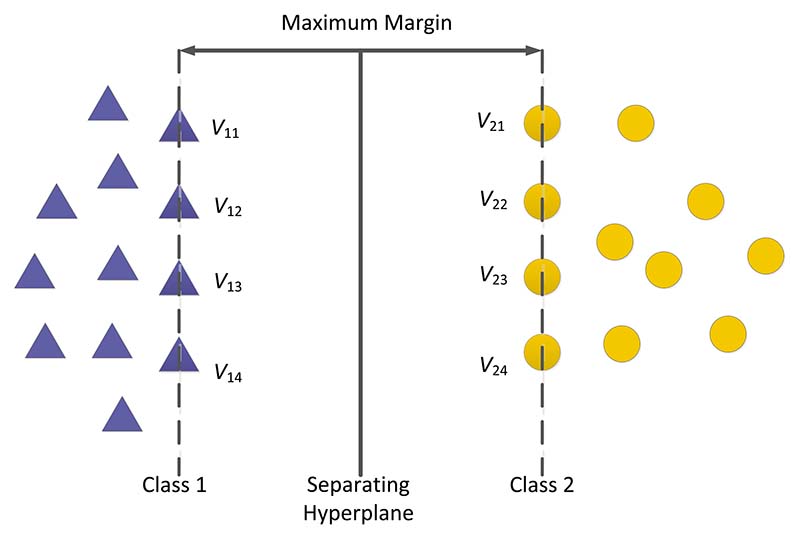
Schematic diagram of linear support vector machine

Therefore, the main idea of the support vector machine is to establish a decision hyperplane and realize the division of two different types of samples by obtaining the maximum distance between two types of samples closest to the plane on both sides of the plane [9], as shown in Fig. 7. Here, *V_ij_* indicates the support vector, and all of which are divided into two categories by the hyperplane.

The model trained by SVM is only related to the support vector, so the algorithm’s complexity is mainly affected by the number of support vectors. Vectors and labels can define the training samples in the two-dimensional feature space. The *N* training samples in the m-dimensional feature space are defined as:

(16) $${\left\{\left(X_{i},y_{i}\right)\right\}}_{i=1}^{N},$$

(17) $$X_{i}=\left[\begin{array}{c} x_{i1}\\ x_{i2}\\ \vdots \\ x_{im} \end{array}\right],y_{i}\in \left\{1,-1\right\}.$$

Here, *X_i_* indicates the *i*th vector of the sample space, *y_i_* indicates the category of the *i*th sample. If the training sample is linearly separable, we describe the hyperplane by the following equation [64]:

(18) $$W^{T}\cdot X+b=0$$

where *w* = {*w*_1_; *w*_2_; …; *w_m_*} is the normal vector of the hyperplane, which determines the direction of the hyperplane. *X* = {*x*_1_; *x*_2_; …; *x_m_*} is the training samples. *T* is the transpose, *b* refers to the biases, which determine the distance between the hyperplane and the origin of the space. Once the normal vector *w* and the biases *b* are determined, a partition hyperplane can be uniquely determined. The distance *d* from the vector *X_i_* to the hyperplane can be calculated by the following formula:

(19) $$d=\frac{\left|w_{1}*x_{1}+w_{2}*x_{2}+\ldots w_{m}*x_{m}+b\right|}{\sqrt{w_{1}^{2}+w_{2}^{2}+\ldots w_{m}^{2}}}=\frac{\left|w^{T}\cdot X+b\right|}{\left\|w\right\|}.$$

We assume that the hyperplane can classify the training samples correctly so that the following relation holds [65]:

(20) $$\{\begin{array}{ll} w^{T}\cdot X_{i}+b\geq +1, & y_{i}=+1\\ w^{T}\cdot X_{i}+b\leq -1, & y_{i}=-1 \end{array}.$$

Here, we define the category label of the points on and above the plane *w^T^* · *X_i_*+*b* = 1 as “+1”, and the category label of the points on and below the plane *w^T^* · *X_i_* + *b* =−1 as “−1”. It can be obtained that the distance *d* between the plane *w^T^* · *X_i_* + *b* = 1, and *w^T^* · *X_i_* + *b* =−1 is

(21) $$d=\frac{2}{\left\|w\right\|}.$$

Here, the distance *d* is the sum of the distances of the two outlier support vectors to the hyperplane and is called the margin. We need to find the segmentation hyperplane with the maximum marginal value, that is, the parameters *w* and *b* (Eq. (20)), satisfying the constraint conditions to maximize *d*.

In practice, the samples are often linearly inseparable, so it is necessary to transform the nonlinear separability into linear separability. In support vector machines, the kernel function can map samples from low-dimensional to high-dimensional space so that SVM can deal with nonlinear problems. In other words, the kernel function extends linear SVM to nonlinear SVM, which makes SVM more universal.

Different kernel functions correspond to different mapping methods. The SVM algorithm was initially used to deal with binary classification problems and extended on this basis. It can also deal with multiple classification problems and regression problems.

#### 3.4.2 GMM

The Gaussian mixture model (GMM) uses the Gaussian probability density functions (normal distribution curves) to quantify the variable distribution accurately and decomposes the distribution of variables into several statistical models based on Gaussian probability density functions (normal distribution curves). Theoretically, suppose the number of Gaussian models fused by a GMM is enough, and the weights between them are set reasonably enough. In that case, the GMM can fit samples with any arbitrary distribution.

Suppose that the Gaussian mixture model consists of *M* Gaussian models, and each Gaussian is called a “Component”, the probability density function of GMM is as follows [66,67]:

(22) $$p\left(x\right)=\sum_{m=1}^{M}p\left(m\right)p\left(x|m\right)=\sum_{m=1}^{M}\pi_{m}N\left(x|\mu_{m},\Sigma_{m}\right),$$

where *x* denotes a D-dimensional feature vector, *p* (*x*|*m*) = *N*(*x*|*μ_m_*, Σ_*m*_) is the probability density function of the *m*th Gaussian model, which can be seen as the probability of *x* produced by the *m*th Gaussian model after selection, as shown in the following formula:

(23) $$N\left(x|\mu ,\Sigma \right)=\frac{1}{{\left(2\pi \right)}^{D/2}}\frac{1}{{\left|\Sigma \right|}^{1/2}}exp\left\{-\frac{1}{2}{\left(x-\mu \right)}^{T}\Sigma^{-1}\left(x-\mu \right)\right\}.$$

**Figure 8:**
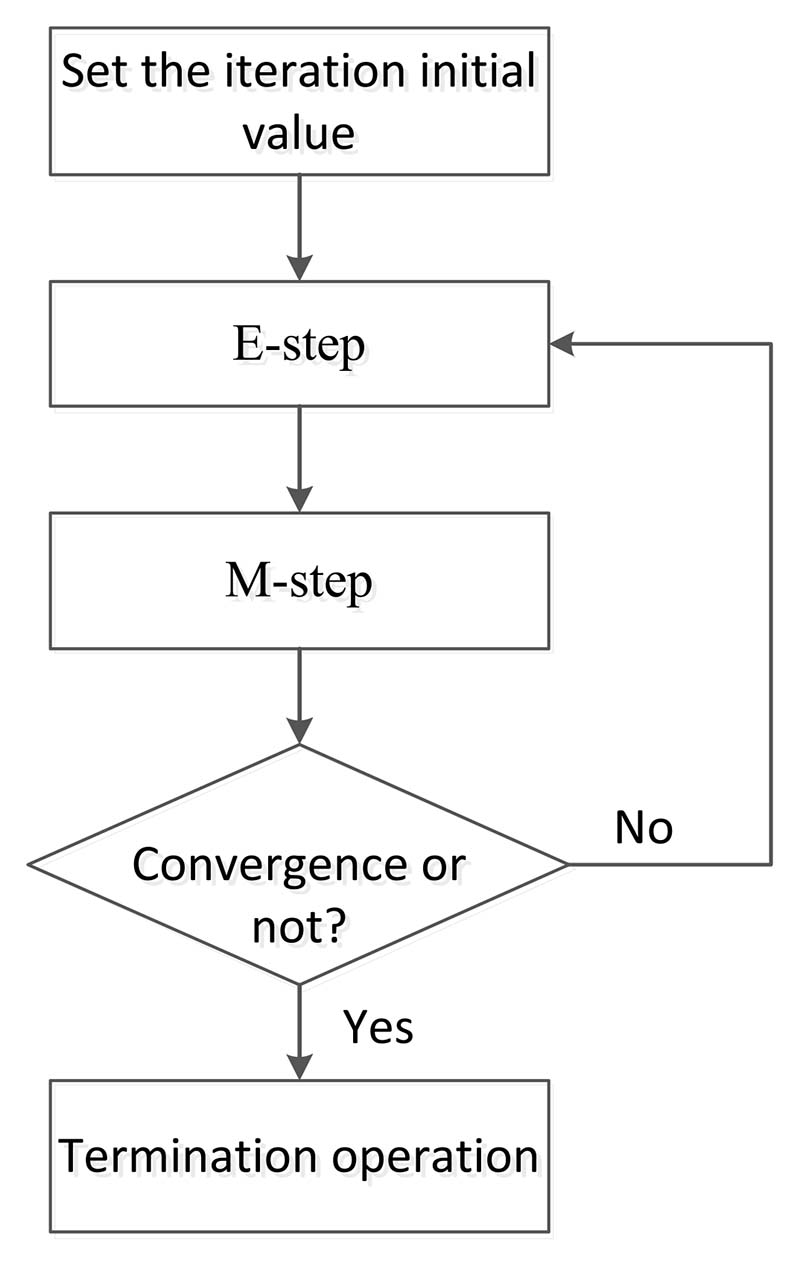
The solving process of GMM based on the EM algorithm

Here, *p* (*m*) = *π_m_* is the weight of the *m*th Gaussian model, that is, the prior probability of choosing the *m*th Gaussian model, and satisfies $\sum_{m=1}^{M}\pi_{m}=1$. Σ represents the covariance of each component, and *μ* represents the average value of each component. Solving the GMM model is essentially to solve these three parameters. The EM algorithm is usually used to solve this problem, which includes expectation-step (E-step) and maximization-step (M-step), as shown in Fig. 8.

(1) E-step
  First, estimate the probability that each component generates the data. Here, we mark the probability of data *x_i_* generated by the *m*th component as *γ* (*i*, *m*), as shown in formula (24).
  (24) $$\gamma \left(i,m\right)=\frac{\pi_{m}N\left(x_{i}|\mu_{m},\Sigma_{m}\right)}{\sum_{j=1}^{M}\pi_{j}N\left(x_{i}|\mu_{j},\Sigma_{j}\right)}.$$
(2) M-step
  Next, iteratively solve the parameter values according to the calculation results of the previous step.
  (25) $$\mu_{m}=\frac{1}{N_{m}}\sum_{i=1}^{N}\gamma \left(i,m\right)x_{i},$$
  (26) $$\sum_{m}=\frac{1}{N_{m}}\sum_{i=1}^{N}\gamma \left(i,m\right)\left(x_{i}-\mu_{m}\right){\left(x_{i}-\mu_{m}\right)}^{T},$$
  (27) $$\pi_{m}=\frac{N_{m}}{N},$$
  where $N_{m}=\sum_{i=1}^{N}\gamma \left(i,m\right)$, repeat the above E-M steps until the value of the log-likelihood function (formula (23)) no longer changes significantly.
  (28) $$lnp\left(x|\pi ,\mu ,\Sigma \right)=\sum_{i=1}^{N}ln\left\{\sum_{m=1}^{M}\pi_{m}N\left(x_{i}|\mu_{m},\Sigma_{m}\right)\right\}.$$

#### 3.4.3 HMM

As we all know, the hidden Markov model (HMM) is a classic machine learning model which has proved its value in language recognition, natural language processing, pattern recognition, and other fields [68,69]. This model describes the process of generating a random sequence of unobservable states from a hidden Markov chain and then generating the observed random sequence from each state. Among them, the transition between the states and the observation sequence and the state sequence have a certain probability relationship [70]. The hidden Markov model is mainly used to model the above process.

We assume that *M* and *N* represent the set of all possible hidden states and the set of all possible observed states, respectively. Then *M* and *N* are expressed as follows:

(29) $$M=\left\{m_{1},m_{2},\ldots ,m_{P}\right\},N=\left\{n_{1},n_{2},\ldots ,n_{Q}\right\},$$

where *P* and *Q* are the number of possible hidden states and the number of possible observed states, respectively, which are not necessarily equal.

**Figure 9:**
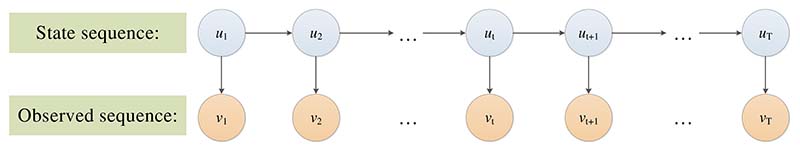
Graph model structure of hidden Markov model

In a sequence of length *T*, *U* and *V* correspond to the state and observation sequences, respectively, as follows:

(30) $$U=\left\{u_{1},u_{2},\ldots ,u_{T}\right\},V=\left\{v_{1},v_{2},\ldots ,v_{T}\right\},$$

where the subscript of each element represents the moment. That is, the state sequence and the observation sequence elements are successively related. Any hidden state *u_t_* ∈ *M* and any observed state *v_t_* ∈ *N*. Therefore, the graph model structure of the above hidden Markov model is shown in the following Fig. 9.

To facilitate the solution, assume that the hidden state at any moment is only related to its previous hidden state. The hidden state at time *t* is *u_t_* = *m_i_* and the hidden state at time *t*+1 is *u*_*t*+1_ = *m_j_*, then the transition probability of HMM state *a_ij_* from time *t* to time *t*+1 can be obtained as follows:

(31) $$a_{ij}=P\left(u_{t+1}=m|u_{i}=m_{i}\right).$$

Thus, the state transition matrix *A* can be obtained:

(32) $$A={\left[a_{ij}\right]}_{P\times P}.$$

Assuming that the observed state at any moment is only related to the hidden state at the current moment when the hidden state at time *t* is *u_t_* = *m_j_* and the corresponding observed state is *v_t_* = *n_k_*, then the probability *b_j_*(*k*) generated by the observed state *n_k_* at this time satisfies the following equation under the hidden state *m_j_*.

(33) $$b_{j}\left(k\right)=P\left(v_{t}=n_{k}|u_{t}=m_{j}\right).$$

In this way, *b_j_*(*k*) can form the probability matrix *B* generated by the observed state.

(34) $$B={\left[b_{j}\left(k\right)\right]}_{P\times Q}.$$

In addition, we define the probability distribution Π of hidden states at time *t* = 1 as follows:

(35) $$\Pi ={\left[\pi \left(i\right)\right]}_{P},$$

where *π* (*i*) = *P*(*u*_1_ = *m_i_*), Π is an n-dimensional vector with each element representing the probability of being in a certain state at time *t* = 1. In this way, the initial probability distribution of hidden states Π, the state transition probability matrix *A*, and the observed state probability matrix *B* can determine the HMM model, which can be expressed as follows [71]:

(36) λ=(A,B,Π).

Here, Π and *A* determines the sequence of states, and *B* determines the sequence of observations.

#### 3.4.4 Other Classification Approaches

In addition to the above classification algorithms (SVM [72–75,43,61], GMM [76,77], HMM [70,69,78]), some other classification algorithms are used for posture recognition, such as k-nearest neighbor (k-NN) [79], random forest (RF) [80–82], Bayesian classification algorithm [83], decision tree (DT) [72,84,85], linear discriminant analysis [86,60], naïve Bayes (NB) [72,87], etc.

## 4 Deep Neural Network-Based Approach

Deep learning mainly uses neural network models, such as convolutional neural network (CNN), deep neural network (DNN), recurrent neural network (RNN), transfer learning, attention model, and long short-term memory (LSTM), as parameter structures to optimize machine learning algorithms.

This method is an end-to-end learning method, which does not require manual operation, but relies on the algorithm to automatically extract features, starting directly from the original input data, and automatically completes feature extraction and model learning through a hierarchical network [17]. In recent years, it has been widely used in many fields and achieved remarkable results, such as image recognition, intelligent monitoring, text recognition, semantic analysis, and other fields. Human posture recognition based on deep learning can quickly fit the human posture information in the sample label so as to generate a model with posture analysis ability.

### 4.1 Posture Estimation

Regarding network architecture, deep learning-based posture estimation is divided into a single-stage approach and a multi-stage approach. The usual difficulty of single-stage networks lies in the subsequent feature fusion work, and multi-stage networks generally repeat and superimpose a small network structure.

Since the number and position of people in the image are unknown in advance, multi-body posture estimation is more difficult than single-body posture estimation, which is usually divided into two ideas: top-down and bottom-up. The former is first to incorporate person detectors, then estimate each part, and finally calculate the pose of each person. The latter is to detect all parts in the image, the parts of each person, and then use a certain algorithm to associate/group the parts belonging to different people. The algorithms mainly include CPM [88], stacked hourglass networks [89], and MSPN [90]. Single-stage approaches are all Top-down, such as CPN [91] and simple baselines [92].

### 4.2 Convolutional Neural Networks

In posture recognition, convolutional neural networks (CNN) have achieved good results. In the system designed by Yan et al. [93], CNN is used to learn and predict the preset driving posture automatically. Wang [94] used CNN to design a human posture recognition model for sports training. CNN has also been successfully used for capture posture detection [95]. Rani et al. [96] adopted the lightweight network of convolution neural network-long short-term memory (CNN-LSTM) for classical dance pose estimation and classification. Zhu et al. [97] proposed a two-flow RGB-D faster R-CNN algorithm to achieve automatic posture recognition of sows, which applied the feature level fusion strategy.

**Figure 10:**
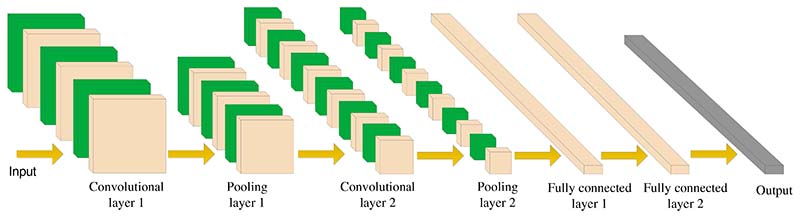
A simple diagram of CNN

The neurons in each layer of convolutional neural networks are arranged in three dimensions (width, height, and depth). It should be noted that depth here refers to the number of layers of the network. The convolutional neural network is mainly composed of the input layer, convolutional layer (CL), ReLU layer, pooling layer (PL), and fully connected layer (FCL). A simple diagram of CNN is shown in Fig. 10.

The core layer of the convolutional neural network is the convolutional layer, which is composed of several convolution units. The important purposes of dimension reduction and feature extraction are achieved through convolution operation. In the first layer of the convolution layer, only some low-level features, such as edges, lines, and angles, can be extracted. In contrast, more complex posture features need to be extracted from more layers of iteration.

The pooling layer is sandwiched between continuous convolution layers to compress the amount of data and parameters, improve identification efficiency and effectively control overfitting. A pooling layer is actually a nonlinear form of drop sampling.

Generally, the full connection layer is in the last few layers and is used to make the final identification judgment. Their activation can be matrix multiplication, and then the deviation is added.

### 4.3 Improved Convolutional Neural Networks

Since the sparse network structure of the traditional CNN cannot retain the high efficiency of dense computation of a fully connected network, and the classification results are inaccurate, or the convergence speed is slow due to the low utilization of convolutional features in the experimental process, so many researchers have carried out various optimization of the CNN algorithm.

For example, by using batch normalization (BN), the distribution of input values of any neuron in each layer of the neural network is forced to return to the normal distribution with a mean of 0 and a variance of 1 (or other), so that the activated input values fall in the sensitive area of the input, thus avoiding the vanishing gradient [98].

Deep residual networks address network degradation using residual learning with identity connections [99]. CNN-LSTM provides solutions to complex problems with large amounts of data [96]. Since target tracking methods based on traditional CNN and correlation filters are usually limited to feature extraction with scale invariance, multi-scale spatio-temporal residual network (MSST-ResNet) can be used to realize multi-scale feature and spatio-temporal interaction between the flows of spatial and time [100], which is also regarded as an extension of residual network architecture. Bounding box regression and labeling from raw images via faster R-CNN showed high reliability [101]. In human posture recognition, many networks based on CNN have emerged (such as stacked hourglass networks, MSPN, CPM, and HRNet [102]).

Stacked hourglass networks show good performance in human posture estimation based on successive pooling and upsampling steps to capture and integrate information at all image scales. The network is combined with intermediate supervision for bottom-up, top-down repetitive processing [89]. The stacked hourglass model is formed by concatenating hourglass modules, each consisting of many residual units, pooling layers, and upsampling layers [103], so it is able to capture all information at each scale and combine these features to output pixel-level predictions.

In the study by Alejandro Newell et al. [89] using a single pipeline with skip layers to preserve spatial information at each resolution, the topology of the hourglass is symmetric. That is, for each layer that exists downward, there is an upper-level corresponding to it. After reaching the output resolution of the network, the final network prediction is completed by two successive rounds of 1 × 1 convolution.

The output of the network is a set of heat maps, and for a given heat map, the probability of a joint occurring at each pixel will be predicted. The remaining modules are used as much as possible in the stacked hourglass network, and local and global features are integrated by each hourglass module, which is further understood in subsequent bottom-up and top-down processing phases. The hourglass modules do not share the weight with each other. The filters are all less than or equal to 3 × 3, and the bottleneck limits the total number of parameters per layer, thus reducing the overall memory usage [89].

Li et al. [90] first introduced the multi-stage pose estimation network (MSPN), which adopted ResNet-based global net as a single-stage module and used a cross-stage feature aggregation strategy, that is, two independent information streams are introduced from the downsampling unit and upsampling unit of the previous stage to the downsampling process of the current stage for each scale, and 1 × 1 convolution is added to each stream for feature aggregation to alleviate the problem of information loss during repeated upsampling and downsampling of multi-stage networks.

Furthermore, feature aggregation can be regarded as an extended residual design that helps solve the vanishing gradient. The multi-stage pose estimation network is designed as a multi-branch supervision method from coarse to fine. Different Gaussian kernel sizes are used at different stages, and the closer the stage kernel-size is to the input, the larger the stage kernel-size will be. Multi-scale supervision is introduced to perform intermediate supervision with four different scales at each stage, resulting in a large amount of contextual information at different levels to help localize challenging poses.

Wei et al. [88] introduced the first pose estimation model based on deep learning, which is called the convolutional pose machine (CPM). CPM combines the advantages of a deep convolutional architecture with a pose machine framework consisting of a series of convolutional networks. In other words, the pose machine’s prediction and image feature calculation modules are replaced by deep convolutional architecture, which allows the image and context features to be directly learned from the data to represent these networks. The convolutional architecture is fully differentiable, and all stages of the CPM can be trained end-to-end. In this way, the problem of structured prediction in computer vision can be solved without inferring the graphical model. Furthermore, the method of intermediate supervision is also used to solve the gradient disappearance problem in the cascade model training process.

The high-resolution network (HRNet) was proposed by Sun et al. [102], showing superior performance in human body pose estimation. This network will connect sub-networks from high resolution to low resolution in parallel to maintain high-resolution expression. Furthermore, the predicted heatmaps are more accurate by performing repeated multi-scale fusions to obtain high-resolution features with low-resolution representations of the same depth and similar levels.

We have introduced several typical CNN-based posture recognition algorithms above, which all have their own characteristics, and the summary is shown in Table 1.

### 4.4 Lightweight Network

The practice proves that a large number of convolutional neural network models have a significant effect on posture recognition. However, with the increasing complexity of convolutional neural network models, the number of layers of the model will gradually deepen accordingly, resulting in an increasing number of parameters, which will require more computing resources. Moreover, with the support of Internet of Things (IoT) technology and smart terminals, such as mobile phones and embedded devices, there is an increasing demand for porting human posture recognition networks to resource-constrained platforms [104]. Therefore, lightweight research on the convolutional neural network model is gradually carried out. The emerging lightweight network models mainly include Squeeze Net [105], Mobile Net [106], Shuffle Net [107], Xception [108], and Shuffle Net V2 [109].

#### 4.4.1 Spatial Separable Convolutions

Spatially separable convolution (SSC) mainly refers to splitting or transforming the convolution kernel, then performing convolution calculations separately, which mainly deals with the two spatial dimensions of image width and height and the convolution kernel. A spatially separable convolution splits a kernel into two smaller kernels.

**Figure 11:**
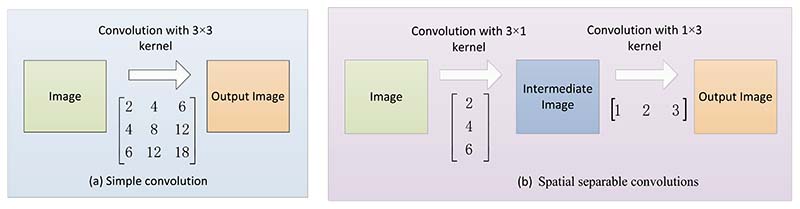
An example of spatial separable convolutions

For example, before a 3 × 3 convolution core is split, nine times multiplication is required to complete a convolution. After being split into a 3 × 1 and 1 × 3 convolution core, three times multiplication is required for each convolution, and a total of 6 multiplications for the combination of the two convolutions can achieve the same effect as before [110], as shown in Fig. 11. The cost of multiplication is reduced, so the computational complexity is reduced, and the network can run faster. It should be noted that not all convolution kernels can be split into two smaller ones.

#### 4.4.2 Depthwise Separable Convolution

In depthwise separable convolution (DSC), one convolution kernel can also be split into two small convolution kernels, but different from spatially separable convolution, depthwise separable convolution can be applied to those convolution kernels that cannot be split, and then perform two calculations for these two convolution kernels: depthwise convolution and pointwise convolution, which greatly reduces the amount of computation in the convolution process.

Depthwise convolution is a channel-to-channel convolution operation that establishes a *k* × *k* convolution kernel for each channel of input data. A convolution kernel convolves a channel, and a channel is convolved only by a convolution kernel. In this process, the number of generated feature mapping channels is exactly equal to the number of input channels [111].

Pointwise convolution operations are very similar to regular convolution operations. A 1 × 1 convolution kernel is implemented on every channel completed by depthwise convolution. The size of the pointwise convolution kernel is 1 × 1 × *L*,where *L* is the number of channels on the upper layer. The mapping in the previous step is weighted in the depth direction by the convolution operation to generate a new feature map pointwise convolution.

The spatial dimension can be processed by depthwise separable convolution, and the matrix can also be divided by the depth of the convolution kernel. It is to segment the channels of the convolution kernel instead of directly decomposing the matrix.

#### 4.4.3 Feature Pyramid Networks

The feature pyramid network (FPN) is designed according to the concept of a feature pyramid. Instead of the feature extractor of detection models (such as faster R-CNN), FPN generates multi-layer feature maps and pays attention to both the texture features of the shallow network and semantic features of the deep network when extracting features.

**Figure 12:**
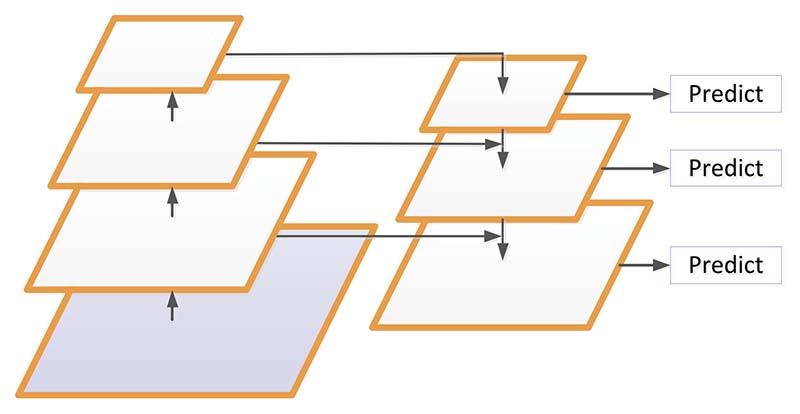
Schematic diagram of FPN network structure

FPN includes three parts: bottom-up path, top-down path, and lateral connection [112], as shown in Fig. 12. The bottom-up path calculation is a feature hierarchy composed of feature maps of multiple scales, which is the traditional convolutional network to achieve feature extraction. With the deepening of the convolution network, the spatial resolution decreases, and the spatial information is lost, but the semantic value of the network layer increases correspondingly and is more detected.

The top-down path builds higher-resolution layers based on semantically richer layers. These features are then augmented by horizontal connections using the features in the bottom-up path [112]. The feature maps of the same spatial size of the bottom-up path and the top-down path are merged by each horizontal connection.

### 4.5 Batch Normalization

For a neural network, the parameters will be continuously updated with the gradient descent, which will cause changes in the data distribution of internal nodes, that is, the internal covariance translation phenomenon. In this case, the above problems can be solved by batch normalization (BN), and the speed of model training and the performance of network generalization can be significantly improved [113].

**Figure 13:**
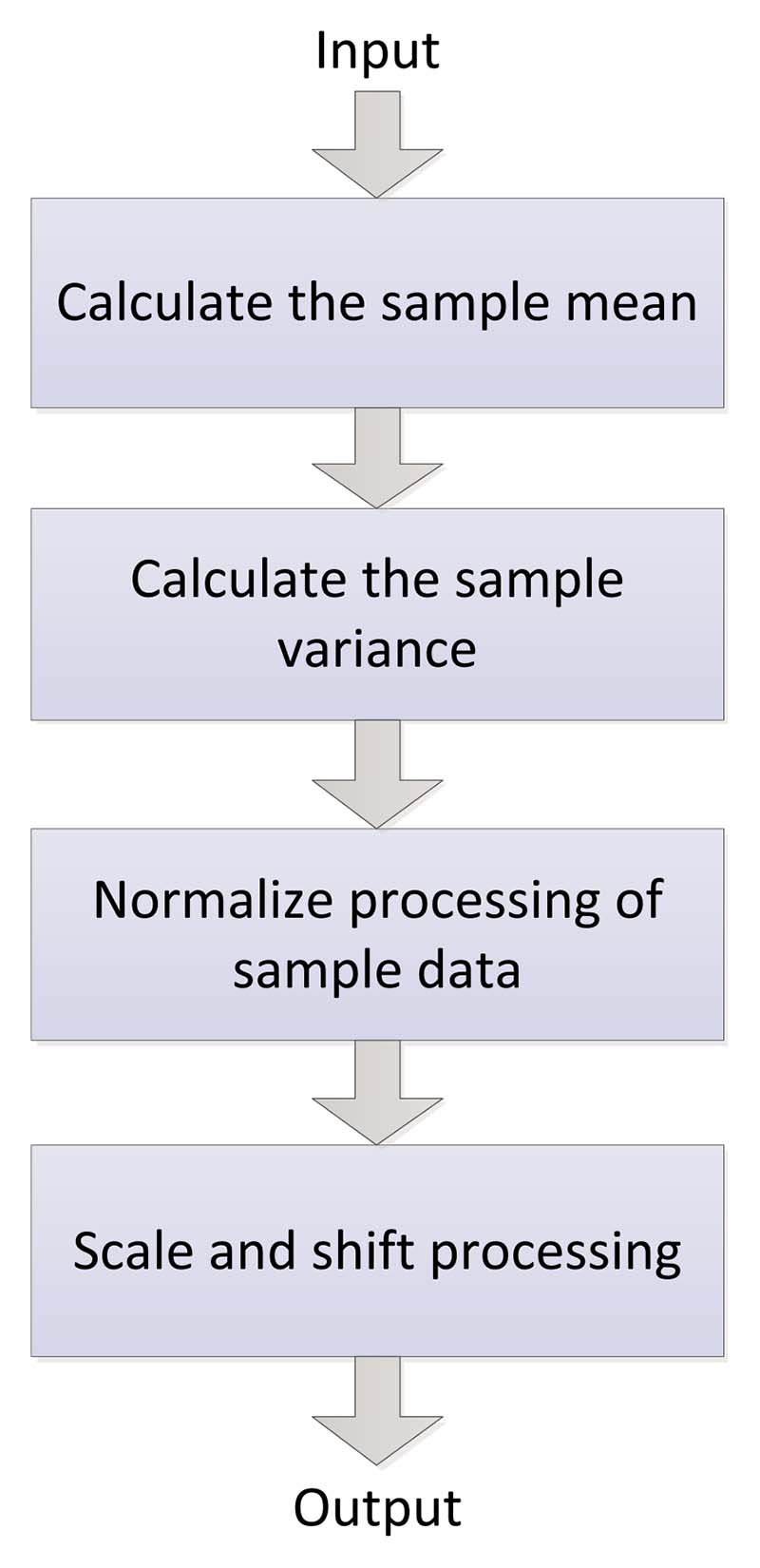
The forward transmission process of the BN network layer

The main idea of BN is that any layer in the network can be normalized, and the normalized feature graph can be re-scaled and shifted to make the data meet or approximate the Gaussian form of distribution. Batch normalization can reparameterize almost any deep network, addressing the situation where the data distribution in the middle layers changes during training [114]. Like the convolution layer, activation function layer, pooling layer, and fully connected layer, batch normalization is also a network layer. The forward transmission process of the BN network layer is shown in Eqs. (37)–(40) and Fig. 13.

(37) $$\mu_{g}=\frac{1}{n}\sum_{i=1}^{n}x_{i},$$

(38) $$\sigma_{g}^{2}=\frac{1}{n}\sum_{i=1}^{n}{\left(x_{i}-\mu_{g}\right)}^{2},$$

(39) $$x_{i}^{\Delta }=\frac{x_{i}-\mu_{g}}{\sqrt{\sigma_{g}^{2}}+\tau },$$

(40) $$BN_{\alpha ,\beta }\left(x_{i}\right)=\alpha x_{i}^{\Delta }+\beta .$$

Here, *μ_g_* refers to mini-batch mean, $\sigma_{g}^{2}$ refers to mini-batch variance, *n* refers to the mini-batch size, and $x_{i}^{\Delta }$ denotes the normalization process. We define *τ* as a very small value to prevent the denominator from being zero. To maintain the expressiveness of the model, we introduce two learning parameters *α* and *β*, *α* refers to the scale factor, and *β* refers to the shift factor.

In convolutional neural networks, batch normalization occurs after the convolution computation and before the activation function is applied. If the convolution calculation outputs multiple channels, the outputs of these channels should be batch normalized separately, and each channel has the independent scale and shift parameters, which are all scalars.

### 4.6 Deep Residual Network

In conventional neural networks, the continuous increase of network depth will lead to the gradual increase of accuracy until saturation and then rapid decline, resulting in the difficulty of deep network training, that is, network degradation, which may be caused by the model being too large and the convergence speed too slow. The degradation problem can be solved by the deep residual network (DRN) [115]. The network layer can be made very deep through this residual network structure, and the final classification effect is also very good.

**Figure 14:**
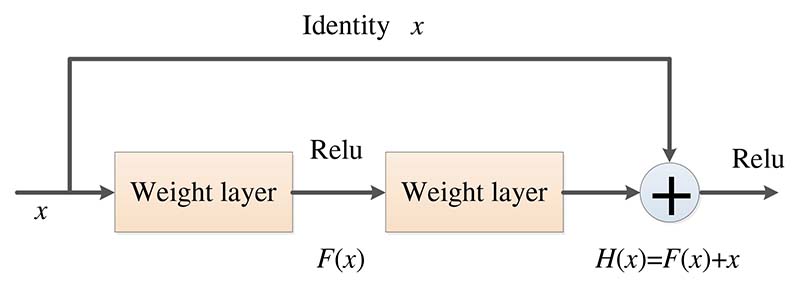
The basic residual block

In the residual network structure, for a neural network with a stacked-layer structure, assuming the input is *x*, *H*(*x*) denotes the learned feature, and the residual that can be learned is expected to be denoted as *F* (*x*) = *H* (*x*) − *x*, so the original learned feature obtained as *F* (*x*) + *x*. *H* (*x*) can be implemented by a feedforward neural network with “shortcut connections” [115], as shown in Fig. 14. When the residual *F* (*x*) is equal to 0, only the identity mapping is completed by the stacking layer, and the goal of the later learning is to approximate the residual result to 0 so that with the deepening of the network, the network performance will not be degraded.

### 4.7 Dropout Technology

In the deep neural network model, if the number of neural network layers is too large, the training samples are few, or the training time is too long, it will lead to the phenomenon of overfitting [116]. Dropout technology can be used to reduce overfitting to prevent complex co-adaptation to training data [117].

In the neural network using dropout technology, a batch of units is randomly selected and temporarily removed from the network at each iteration in the training stage, keeping these units out of forward inference and backward propagation [116].

It should be noted that instead of simply discarding the outputs of some neural units, we need to change the values of the remaining outputs to ensure that the expectations of the outputs before and after discarding remain unchanged. In general, a fixed probability *p* that each cell retains can be selected using the validation set, and the probability *p* is often set to 0.5. Still, the optimal retention probability is usually closer to 1 for input cells. In networks with dropout, the generalization errors of various classification problems can be significantly reduced using the approximate averaging method.

**Figure 15:**
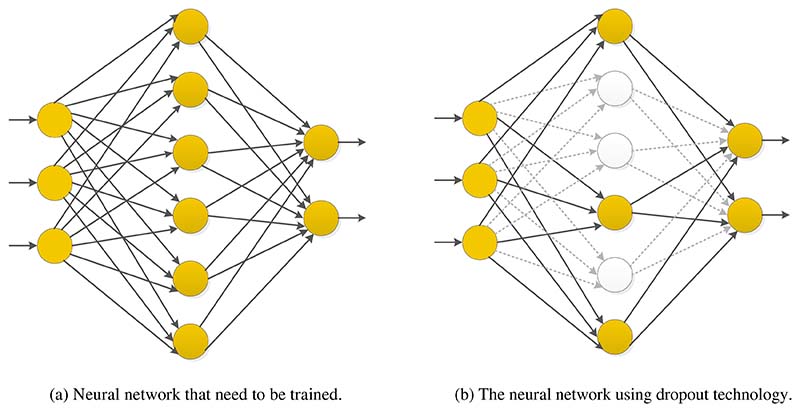
A neural network using dropout technology

Suppose we want to train such a neural network, as shown in Fig. 15a. After Dropout is applied to the neural network, the training process is mainly the following:

(i) Randomly delete half of the hidden neurons in the network. Note that these deleted neurons are only temporarily deleted, not permanently deleted, and the input and output neurons remain unchanged, as shown in Fig. 15b.
(ii) The input is then propagated forward along the modified network, and the loss result is propagated back along the modified network. After this procedure was performed on a small group of training samples, the parameters of the neurons that were not deleted were updated according to the random gradient descent (SGD) method.
(iii) Restore the deleted neuron. At this time, the deleted neuron parameters keep the results before deletion, while the non-deleted neuron parameters have been updated. The above process is repeated continuously.

### 4.8 Advanced Activation Functions

In a neural network, an important purpose of using multi-layer convolution is to use the size of different convolution kernels to extract image features at different convolution kernel scales. The convolution algorithm is composed of a mass of multiplications and additions, so the convolution algorithm is also linear and can be considered a linear weighting operation through the convolution kernel. The convolutional neural network composed of many convolution algorithms will degenerate into a simple linear model without introducing nonlinear factors, making the multi-layer convolution meaningless.

Therefore, adding a nonlinear function after the convolution of each layer of the neural network can complete the linear isolation of the two convolution layers and ensure that each convolution layer completes its own convolution task. Currently, the common activation functions mainly include sigmoid, tanh, rectified linear unit (ReLU), etc. Compared with the traditional activation functions of neural networks, such as sigmoid and tanh, RELU has the following advantages: (i) When the input of the ReLU function is positive, the gradient saturation will not occur in the network. (ii) Since the ReLU function has only a linear relationship, its calculation speed is faster than sigmoid and tanh. The definition of the ReLU function is shown in Eq. (41) [118]:

(41) $$h_{\text{ReLU}}\left(x_{i}\right)=\max\left(0,x_{i}\right),$$

where *x_i_* is the input in the *i*th channel. There are many variants of the ReLU function, such as parametric ReLU, leaky ReLU, random ReLU, etc. Each activation function has advantages in one or several specific deep learning networks.

Leaky ReLU (LReLU) is similar to ReLU, except that the input is less than 0. In the ReLU function, all negative values are zero, and the outputs are non-negative. In contrast, in the Leaky ReLU, all negative values are assigned a non-zero slope with a negative value and a small gradient [119]. The Leaky ReLU activation function can avoid zero gradients, which is defined as follows:

(42) $$h_{L\mathrm{ReLU}}\left(x_{i}\right)=\{\begin{array}{ll} x_{i} & if\;\;\;x_{i}>0\\ \alpha_{i}x_{i}, & if\;\;\;x_{i}\leq 0 \end{array}.$$

Here, *a_i_* is a fixed parameter, usually with a value of 0.01. In the process of backpropagation, the gradient can also be calculated for the part of the Leaky ReLU activation function input less than zero, which can avoid the problem of gradient direction aliasing.

Parametric ReLU (PReLU) adaptively learns to rectify the parameters of linear units and is able to improve classification accuracy at a negligible extra computational cost [120]. The definition of the PReLU function is shown in Eq. (43):

(43) $$h_{\mathrm{PReLU}}\left(x_{i}\right)=\{\begin{array}{ll} x_{i}, & if\;\;\;x_{i}>0\\ \beta_{i}x_{i}, & if\;\;\;x_{i}\leq 0 \end{array}.$$

Here, *β_i_* is responsible for controlling the slope of the negative semi-axis, and the activation functions of different channels can be different. When the value of *β_i_* is 0, PReLU can be regarded as ReLU. If the value of *β_i_* is small and fixed, then PReLU can be considered Leaky ReLU.

Randomized ReLU (RReLU) can be understood as a variant of Leaky ReLU. The definition of RReLU function is shown as follows:

(44) hRReLU(xji)={xji,ifxji>0αijxji,ifxji≤0.

Here, *x_ji_* represents the input of the *i*th channel in the *j*th example, *a_ji_* is a random value drawn from a uniform distribution *U* (*l*, *u*).

(45) $$\alpha_{ij}∼U\left(l,u\right),l<u\;\text{and}\;l,\;\;u\in \left[0,1\right).$$

**Figure 16:**
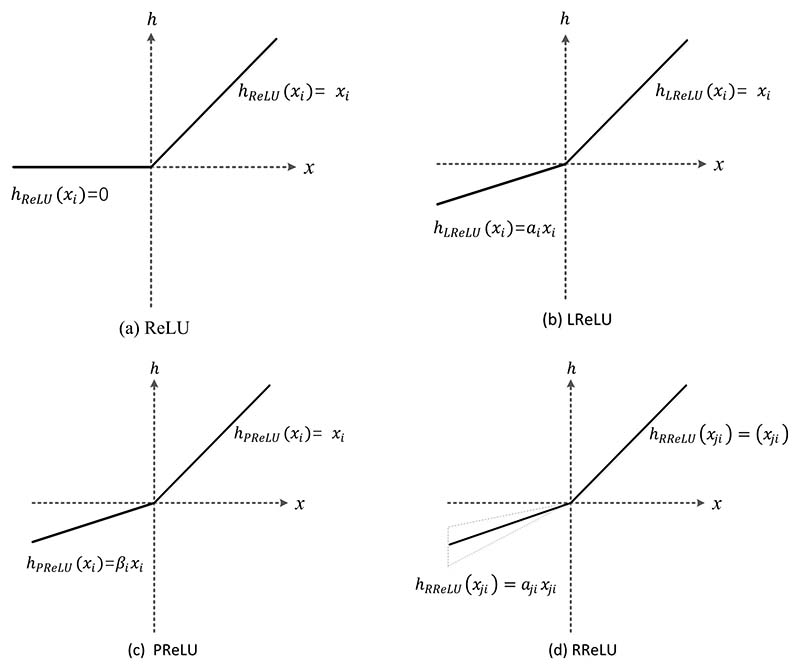
The diagrams of ReLU, LReLU, PReLU, and RReLU

The diagrams of ReLU, LReLU, PReLU, and RReLU are shown in the following Fig. 16 [121].

## 5 Advanced Neural Networks

In order to improve the performance of the system, some advanced neural networks are studied in the field of posture recognition, such as transfer learning, ensemble learning, graph neural networks, explainable deep neural networks, etc.

### 5.1 Transfer Learning

Transfer learning (TL) refers to the transfer of the trained model parameters to the new model to help the new model training [122]. Transfer learning technology has been used in posture recognition. Hu et al. [123] used transfer learning in their sleep posture system, and the system accuracy and real-time processing speed were much higher than the standard training-test method. Ogundokun et al. [124] applied the transfer learning algorithm with hyperparameter optimization (HPO) to human posture detection. The experiments show that the algorithm is superior to the algorithm using image enhancement in terms of training loss and verification accuracy, but the system’s complexity increased after the algorithm was used. Long et al. [125] developed a yoga self-training system using transfer learning techniques.

Considering that most data or tasks are related, through transfer learning, we can share the learned model parameters with the new model in some way to speed up and optimize the learning efficiency of the model. It is one of the advantages of transfer learning that we do not need to learn from zero like most networks. In addition, in the case of small data sets, transfer learning can get good results, and we can also use transfer learning to reduce training cost sets.

### 5.2 Ensemble Learning

Ensemble learning (EL) is to construct and combine multiple machine learning machines to complete learning tasks. The process generates a group of “individual learning machines” and then combines them with a certain strategy [126]. Individual learning machines are common machine learning algorithms, such as decision trees and neural networks [127]. Ensemble learning can be used for classification problem integration, regression problem integration, feature selection integration, outlier detection integration, and so on.

Ensemble learning is used in sensor-based posture recognition systems to overcome the problems of data imbalance, instant recognition, sensor deployment, and selection when collecting data with wearable devices [128]. Liang et al. [129] designed a sitting posture recognition system using an ensemble learning classification model to ensure the generalization ability of the system. Esmaeili et al. [130] designed a posture recognition integrated model by superimposing two classification layers based on the deep convolution method.

### 5.3 Graph Neural Networks

Graph neural networks (GNN) is a framework that uses deep learning to learn the graph structure data directly. Aggregating features of adjacent nodes calculate the features of each node, and the graph dependency is established by passing messages between nodes [131]. In GNN, graph properties (such as points, edges, and global information) are transformed without changing the connectivity of the graph.

GNN has achieved excellent results in posture recognition tasks. Guo [132] formed a multi-person posture estimation algorithm based on a graph neural network by using multilevel feature maps, which greatly improved the positioning accuracy of each part of the human body. Li et al. [133] used the graph neural network to optimize posture graphs, which achieves good efficiency and robustness. Taiana et al. [134] constructed a system based on graph neural networks that can produce accurate relative poses.

In recent years, the variants of GNN variants, such as graph convolutional networks (GCNs), graph attention networks (GATs), and gated graph neural networks (GGNNs), have shown breakthrough performance in many posture recognition tasks [135–137].

## 6 Analysis and Discussion

We have detailedly reviewed the techniques and methods of posture recognition, including the process of posture recognition, feature extraction, and classification techniques. Compared with the existing reviews in recent years, this paper presents the following advantages: (i) This paper combs the pose recognition technologies and methods based on traditional machine learning and deep learning-based posture recognition technologies and methods and summarizes and analyzes 2D and 3D datasets, which is more comprehensive in content; (ii) In order to timely share the latest technologies and methods of posture recognition with readers, this review focuses on the latest development of posture recognition technologies and methods. The literature on posture recognition technologies and methods is relatively new, and most of them are related research papers from the past five years, which have been updated in time. We list the comparison of recent reviews on posture recognition as shown in Table 2.

To help understand more clearly, we created a table of abbreviations and corresponding full names for posture recognition terms as follows (Table 3):

### 6.1 Main Recognition Techniques

According to data acquisition, posture recognition technology is divided into sensor-based recognition technology, vision-based recognition technology, and RF-based recognition technology.

Sensor-based recognition methods are less costly and simple to operate but are limited to devices and require the real-time wearing of sensors [17,145].

Vision-based recognition method has high accuracy and overcomes the problem of wearing. It is easy to obtain the trajectory, contour, and other information about human movement. However, this method is affected by light, background environment, and other factors and is prone to recognition errors due to occlusion and privacy exposure [146,147].

RF-based identification technology has the characteristics of non-contact and is very sensitive to environmental changes. It is easily affected by the human body’s absorption, reflection, and scattering of RF signals [31]. The characteristics of the three recognition methods are shown in Table 4.

### 6.2 2D Posture Recognition and 3D Posture Recognition

According to the difference in human posture dimensions, the human posture recognition task can be divided into two-dimensional human posture recognition and three-dimensional human posture recognition. The purpose of two-dimensional human posture recognition is to locate and identify the keypoints of the human body. Then these key points are connected in the order of joints, which are projected on the two-dimensional plane of the image to form the human skeleton.

There are currently many 2D recognition algorithms, and the accuracy and processing speed have been greatly improved. However, the keypoints of 2D are greatly affected by wearing, posture and perspective. They are also affected by the environment, such as occlusion, illumination, and fog, which require high requirements for data annotation. In addition, the keypoints of 2D are not easy to estimate the positions between human body parts through vision.

3D posture recognition can give images a more stable and understandable interpretation. In recognition of human 3D posture, the 3D coordinate position and angle of human joints are mainly predicted. We can use the 3D posture estimator to convert objects in the image into 3D objects by adding depth to the prediction, that is, to realize the mapping between 2D keypoints and 3D keypoints. There are two specific methods: One is to directly regress 3D coordinates from 2D images [148,149], and the other is to obtain the data of 2D first and then “lift” to 3D posture [150,151].

In 3D posture recognition, due to the addition of depth information on the basis of 2D posture recognition, the expression of human posture is more accurate than in 2D, but there will be occlusion, and it also faces challenges such as the inherent deep ambiguity and inadequacy in single-view 2D to 3D mapping, and the lack of large outdoor datasets. Currently, the mainstream datasets are established in the laboratory environment, and the model’s generalization ability is weak. In addition, there is a lack of special posture datasets, such as falling and rolling.

### 6.3 Recognition Based on Traditional Machine Learning and Deep Neural Network

Traditional machine learning-based recognition methods mainly describe and infer human posture based on the human body models and extract image posture features through algorithms, which have high requirements on feature representation and spatial position relationship of keypoints. Excluding low level features (such as boundary and color), typical high-level features, such as scale-invariant feature transformation and gradient histogram, have stronger expression ability and can effectively compress the spatial dimension of features, showing advantages in terms of time efficiency.

Posture recognition based on deep learning can be trained and learned through the image data of the network model, and the most effective representation method can be directly obtained. The core of posture recognition based on deep learning is the depth of neural networks. Semantic information is extracted from the image through a convolutional neural network, richer and more accurate and reflects better robustness than artificial features.

Moreover, the expressive ability of the network model will increase exponentially with the increase of the network stack number. However, overcoming factors such as occlusion, inadequate training data, and depth blur is still difficult. The commonly used posture recognition algorithms [143,152] in recent years are shown in Table 5.

### 6.4 Datasets

In the field of posture recognition, the successful application of deep learning has significantly improved the accuracy and generalization ability of two-dimensional posture recognition, where the datasets play a crucial role in the system [143]. We list widely used 2D posture benchmark datasets, as shown in Table 6.

Compared with 2D posture recognition, 3D posture recognition faces more challenges, among which deep learning algorithms rely on huge training data. However, due to the difficulty and high cost of 3D posture labeling, the current mainstream datasets are collected in the laboratory environment and lack large outdoor datasets. This will inevitably affect the generalization performance of the algorithm on outdoor data [138,143]. The widely used 3D posture recognition datasets are shown in Table 7.

### 6.5 Current Research Direction

At present, posture recognition is divided into the following research directions:

(1) Pose machines. The pose machine is a mature 2D human posture recognition method. In order to make use of the excellent image feature extraction ability of the convolutional neural network, the convolutional neural network is integrated into the framework of the pose machine [88].
(2) Convolutional network structure. In recent years, significant progress has been made in posture recognition based on convolutional network structure, but there is still room for optimization in recognition performance. Many researchers focused on the optimization of convolutional network structures, and some optimization models were proposed, such as stacked hourglass network [89], iterative error feedback (IEF) [154], Mask R-CNN [156], and the EfficientPose [169].
(3) Multi-person posture recognition in natural scenes. Due to many factors, such as complex background, occlusive congestion, and posture difference in the natural environment, many posture recognition methods with a fine performance in the experimental environment are ineffective in multi-person posture recognition tasks. However, with the development of the field of posture recognition, multi-person recognition in natural scenes is very worthy of study. Fortunately, the recognition of multiple people in natural scenes has attracted the attention of many scholars [155].
(4) Attention mechanism. By designing different attention mechanism characteristics for each part of the human body, more accurate human posture recognition results can be obtained. Some attention-related strategies have been proposed, for example, the attention regularization loss based on local feature identity to constrain attention weight [135], the convolutional neural network with multi-context attention mechanism is incorporated into the end-to-end framework of posture recognition [159], and the polarization self-attention block is realized through polarization filtering and enhancement techniques [168].
(5) Data fusion. The performance of the data fusion algorithm directly affects the accuracy of posture recognition and the reliability of the system [196,197]. Data fusion strategies include multi-sensor-based data fusion [17,198,199], position and posture-based fusion [200], multi-feature fusion [201,202], and so on.

## 7 Conclusion and Future Directions

**Figure 17:**
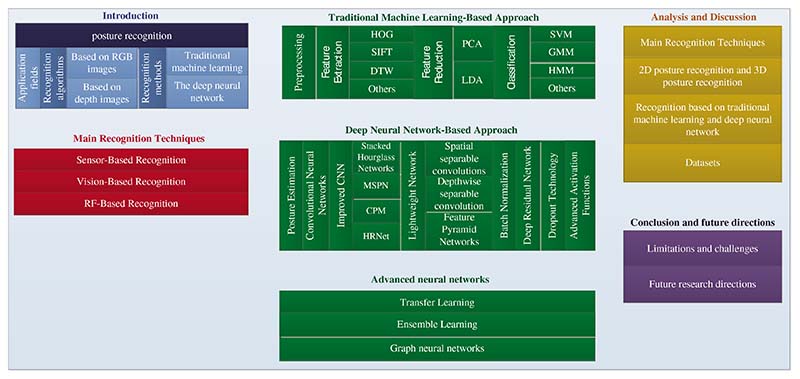
The systematic diagram of our study

This paper reviews and summarizes the methods and techniques of posture recognition. It mainly includes the following aspects: (i) The structure and related algorithms based on traditional machine learning and deep neural network are presented; (ii) The background and application of three posture recognition techniques are presented, and their characteristics are compared; (iii) Several common posture recognition network structures based on CNN are presented and compared; (iv) Three typical lightweight network design methods are presented; (v) The commonly used datasets for posture recognition are summarized, and the limitations of 2D and 3D datasets are talked about. In summary, the framework of our review is shown in Fig. 17.

### 7.1 Limitations and Challenges

Although the techniques and methods of posture recognition have made great progress in recent years, posture research will still face challenges due to the complexity of the task and the different requirements of different fields. Through the research, we believe that the challenges facing posture recognition at this stage mainly include the following aspects, as shown in Table 8.

(1) Datasets problems
  (i) Lack of special posture datasets. Currently, the existing public datasets have a large amount of data, but most of the human posture is normal, such as standing, walking, and so on. Lack of special postures, such as falling, crowding, etc.
  (ii) Lack of large outdoor 3D datasets. The production of 3D posture datasets mostly relies on motion capture equipment, which has restrictions on the environment and the range of human activity, so 3D datasets in outdoor scenes are relatively scarce.
(2) Poor generalization ability
  Since many datasets are established in the experimental environment, the generalization ability of the human posture recognition model in natural scenes is poor, and it is difficult to achieve an accurate posture recognition effect in practical applications [203].
(3) Human body occlusion problem
  Human body occlusion is one of the most important problems in the process of posture recognition, especially in the natural environment of multi-person posture recognition. Human body occlusion is very common. The phenomenon of human-body occlusion includes the occlusion of the human body itself, the occlusion of other objects on the human body, and the occlusion of other human bodies on the human body [204,205]. The occlusion of the human body has a great influence on the prediction of human body joints.
(4) The contradiction between model accuracy and computational power and large storage space
  Deep learning algorithm has become the mainstream method of posture recognition. Many existing posture recognition technologies based on deep learning blindly pursue accuracy, and the design of complex and multi-level networks leads to high requirements on hardware, which is not good for the wide application of neural networks. Therefore, it is particularly important to carry out lightweight design on the network while maintaining recognition accuracy.
(5) Depth ambiguity problem
  Depth ambiguity is a problem in 3D posture recognition, which may result in multiple 3D postures corresponding to the same 2D projection. Additional information needs to be added by the algorithm to recover the correct 3D posture [206]. Many approaches attempt to solve this problem by using a variety of prior information, such as geometric prior knowledge, statistical models, and temporal smoothness [207]. However, there are still some unsolved challenges and gaps between research and practical application.

### 7.2 Future Research Directions

In the future, the research of posture recognition can proceed from the following two aspects of the above discussion of the challenges. (i) Establish an appropriate posture benchmark database, which can be integrated and improved. (ii) The technology based on CNN and other deep neural networks have the potential for improvement, which can be researched in feature extraction, information fusion, and other aspects. (iii) The robustness and stability of body mesh reconstruction under heavy occlusion need to be further explored [208]. (iv) Lightweight network design can be used to solve the contradiction between model accuracy, computing power, and large storage space. It still has a lot of room for improvement in recognition accuracy.

## Figures and Tables

**Table 1:** Summary of several improved CNN algorithms

Improved CNN	Description	Dataset	Performances	Characteristics
Newell et al. [89]	Stacked hourglass networks	FLIC	PCK@0.2: elbow: 99.0%, Wrist: 97%	The bottom-up and top-down structures are repeatedly used in the network architecture, using intermediate supervised learning, and the network converges quickly. The mechanism is simple and can handle diverse and challenging pose sets. Heavy shielding and close contact with multiple people will lead to ambiguity or even overlap.
		MPII	PCKh@0.5: 90.9% (Total)	
Li et al. [90]	MSPN	COCO	Single model: 76.1 AP, ensemble model: 78.1 AP	Multi-stage pipeline with a single-stage module, supervision from coarse to fine. The Cross-stage feature aggregation strategy is used to reduce the information loss and realize the multi-person posture estimation.
		MPII	PCKh@0.5: 92.6% (Mean)	
Wei et al. [88]	CPM	MPII	PCKh@0.5: 87.95% (Total)	Predict the long-term dependencies between variables in a structured task with an implicit model. The accuracy of part location is improved. Close multi-person processing and a single end-to-end architecture are less efficient.
		LSP	PCK: 84.32%	
		FLIC	PCK@0.2:elbow: 97.59%, Wrist: 95.03%	
Sun et al. [102]	HRNet	COCO	HRNet-W48: 75.5 AP, HRNet-W48 + extra data: 77.0 AP	The whole process is represented by high resolution, and the multi-resolution representation is repeatedly fused to present a reliable high-resolution representation.
		MPII	PCKh@0:5: Single-scale testing: 90.3% (Total) Multi-scale testing: 90.8% (Total)	

**Table 2:** The comparison of recent reviews on posture recognition

References	Year	Focus
[138]	2020	Monocular 3D human pose estimation
[139]	2021	Monocular multi-person pose estimation
[140]	2021	3D human pose estimation algorithms for markerless motion capture
[141]	2021	2D multi-person pose estimation methods
[142]	2021	Deep 3D human pose estimation
[143]	2021	Human pose estimation and its application to action recognition
[144]	2022	The application of hardware technology in the posture recognition system

**Table 3:** The list of all abbreviations and full names

Abbreviations	Full names
AI	Artificial intelligence
ANN	Artificial neural network
AP	Average precision
BN	Batch normalization
CACT	Cascade-adaboosting-CART
CFN	Coarse-fine network
CL	Convolutional layer
CNN	Convolutional neural network
CPM	Convolutional pose machine
CPN	Cascaded pyramid network
CRF	Conditional random field
DA	Data augmentation
DNN	Deep neural network
DoG	Difference of Gaussian
DRN	Deep residual network
DSC	Depthwise separable convolution
DT	Decision tree
DTW	Dynamic time warping
DWT	Discrete wavelet transform
ECG	Electrocardiogram
EEG	Electroencephalogram
EL	Ensemble learning
EMG	Electromyogram
FCL	Fully connected layer
FD	Fourier descriptor
FPN	Feature pyramid network
GAN	Generative adversarial network
GATs	Graph attention networks
GCNs	Graph convolutional networks
GGNNs	Gated graph neural networks
GLCM	Gray-level co-occurrence matrix
GMM	Gaussian mixture model
GNN	Graph neural networks
GSR	Galvanic skin response
HAR	Human activity recognition
HMI	Hu moment invariant
HMM	Hidden Markov model
HMR	Human mesh recovery
HOD	Histogram of oriented displacement
HPO	Hyperparameter optimization
HOG	Histogram of oriented gradients
HRNet	High-resolution net
HSV	Hue saturation value
IEF	Iterative error feedback
IMU	Inertial measurement unit
IoT	Internet of things
k-NN	k-nearest neighbor
LDA	Linear discriminant analysis
LMC	Leap motion controller
LoG	Laplacian of Gaussian
LReLU	Leaky rectified linear unit
LSTM	Long short-term memory
MPJPE	Mean per joint position error
MPR	Multi-pose recognition
MSPN	Multi-stage pose estimation network
MSST-ResNet	Multi-scale spatio-temporal residual network
NBC	Naive Bayes classifier
NWFE	Nonparametric weighted feature extraction
PCA	Principal component analysis
PL	Pooling layer
PReLU	Parametric rectified linear unit
PRN	Pose residual network
R-CNN	Region-CNN
ReLU	Rectified linear unit
ResNet	Residual neural network
RF	Random forest
RFID	Radio frequency identification
RMPE	Regional multi-person pose estimation
RNN	Recurrent neural network
RReLU	Randomized leaky rectified linear unit
SCGA	Squeezed convolutional gated attention
SGD	Stochastic gradient descent
SIFT	Scale-invariant feature transform
SSC	Spatially separable convolution
SVM	Support vector machine
TL	Transfer learning
VGG	Visual geometry group
VHMM	Validation hidden Markov model
WE	Wavelet entropy
WVS	Wireless visual sensor

**Table 4:** Summary of main recognition techniques for posture

Method	Technology	Advantages	Disadvantages
Sensor-based	Smartphone, accelerometer, gyroscope	Low cost	Constrains of carrying device
Vision-based	Camera	High accuracy	High cost, complex computation, privacy issue
RF-based	Wi-Fi	Cost-effective, Widely available	Environmental disturbance, unable to provide fine-grained recognition
	RFID	Cost-effective, Widely available	Environmental disturbance
	Radar	Widely available	Environmental disturbance, unable to provide fine-grained recognition

**Table 5:** Common algorithms for posture recognition research

References	Method	Year	Datasets	Accuracy/performance	Characteristics
Pishchulin et al. [153]	DeepCut	2016	MPII	54.10% (pckh-0.5)	Bottom-up
Pishchulin et al. [153]	DeeperCut	2016	MPII	59.40% (pckh-0.5)	Bottom-up
Wei et al. [88]	CPM	2016	MPII	87.95% (pckh-0.5)	Bottom-up
Newell et al. [89]	Stacked hourglass networks	2016	MPII	90.90% (pckh-0.5)	Bottom-up
Carreira et al. [154]	IEF	2016	MPII	81.3% (pckh-0.5)	Single-person
Fang et al. [155]	RMPE	2017	MS COCO	61.80% (AP)	Top-down
He et al. [156]	Mask R-CNN	2017	MS COCO	63.10% (AP)	Top-down
Newell et al. [157]	Associative embedding	2017	MS COCO	65.50% (AP)	Bottom-up
Huang et al. [158]	CFN	2017	MS COCO	72.60% (AP)	Top-down
Newell et al. [157]	Associative embedding	2017	MPII	77.50% (mAP)	Bottom-up
Fang et al. [155]	RMPE	2017	MPII	82.10% (pckh-0.5)	Top-down
Chu et al. [159]	CRF	2017	MPII	91.50% (pckh-0.5)	Bottom-up
Fang et al. [155]	AlphaPose	2017	MPII	76.7% (mAP-0.5)	Top-down
Fang et al. [155]	AlphaPose	2017	MS COCO	71.0 (AP)	Top-down
Kocabas et al. [160]	PRN	2018	MS COCO	69.60% (AP)	Bottom-up
Chen et al. [91]	CPN	2018	MS COCO	73.00% (AP)	Top-down
Xiao et al. [92]	Simple baseline	2018	MS COCO	73.70% (AP)	Top-down
Kanazawa et al. [161]	HMR	2018	Human3.6M	56.80 mm (average MPJPE)	Bottom-up
Kocabas et al. [160]	MultiPoseNet	2018	MS COCO	70.5 (AP)	Bottom-up
Kreiss et al. [162]	PifPaf	2019	MS COCO	66.70% (AP)	Top-down
Li et al. [90]	MSPN	2019	MS COCO	76.10% (AP)	Top-down
Sun et al. [102]	HRNet-W48	2019	MS COCO	77.00% (AP)	Bottom-up
Sun et al. [102]	HRNet-W48	2019	MPII	90.80% (pckh-0.5)	Bottom-up
Xu et al. [163]	DenseRaC	2019	Human3.6M	48.00 mm (average MPJPE)	Bottom-up
Zhao et al. [137]	SemGCN	2019	Human3.6M	43.80 mm (average MPJPE)	Bottom-up
Gujjar et al. [164]	Res-EnDec	2019	JAAD	81.14% (AP)	deep learning
Huang et al. [165]	DeepFuse	2020	Human3.6M	37.50 mm (average MPJPE)	Bottom-up
Zhong et al. [166]	SocialGAN	2020	3D Pedstria Trajectory	71.60% (prediction error)	Bottom-up
Cao et al. [167]	OpenPose	2021	MS COCO	60.50% (AP)	Bottom-up
Liu et al. [168]	UDP-Pose-PSA	2021	MS COCO	79.50% (AP)	Bottom-up
Cao et al. [167]	OpenPose	2021	MPII	76.50% (AP)	Bottom-up
Groos et al. [169]	EfficientPose IV	2021	MPII	91.20% (pckh-0.5)	Bottom-up
Shan et al. [170]	Pose3D-RIE	2021	Human3.6M	30.10 mm (average MPJPE)	Bottom-up
Reddy et al. [171]	TesseTrack	2021	Human3.6M	18.70 mm (average MPJPE)	Bottom-up
Yau et al. [172]	Graph-SIM	2021	PePScenes	94.40% (accuracy)	Deep learning

**Table 6:** Datasets for 2D human posture recognition (I = Image, V = Video, S = Single-person, M = Multi-person)

Dataset	Year	Data source	Single/Multi person	#Keypoints	#Train	#Test
LSP [173]	2010	I	S	14	1,000 images	1,000 images
LSP extended [174]	2011	I	S	14	10,000 images	-
FashionPose [175]	2013	I	S	13	6,530 images	1,000 images
J-HMDB [176]	2013	V	S	13	31,838 frames	-
FLIC [177]	2013	I	S	10	3,987 images	1,016 images
Penn Action [178]	2013	V	S	13	1,163 videos	1,163 videos
MPII [179]	2014	I	S	16	28,821 images (40,522 people)	11,701 images
MPII (Multi-person) [179]	2014	I	M	16	3,844 images	1,758 images
MSCOCO Keypoints [180]	2014	I	M	17	64,115 images (262,465 people)	40,670 images (test-std)
						20,288 images (test-dev)
AI challenger [181]	2017	I	M	14	210,000 images	30,000 images
PoseTrack [182]	2017	V	M	14	20 videos	20 videos
PoseTrack [183]	2018	V	M	15	292 videos	208 videos
CrowdPose [184]	2019	I	M	14	10,000 images	8,000 images
Human-in-Events (HiEve) [185]	2020	V	M	14	49,820 frames (1,099,357 people)	—

**Table 7:** Datasets for 3D human posture recognition (S = Single-person, M = Multi-person)

Dataset	Year	#Frame #Video Sequence	Size/Characters	Single/Multi-person
HumanEva-I&II [186]	2010	80,000	4 subjects, lab environment	S
		56		
Human3.6M [187]	2014	3.6 millions	About 3.6 × 106 poses, lab environment	S
		1 376		
CMU Panoptic [188]	2015	1.5 million	Large scale, multiple perspectives, multiple people	M
		65		
Joint Track Auto (JTA) [189]	2016	460,800	Contains high-definition videos of pedestrians walking in urban scenes	M
		512		
MPI-INF-3DHP [190]	2017	1.3 millions	8 subjects, indoor & outdoor	S
		64		
SURREAL [191]	2017	6 million	The texture SMPL model on the background image is rendered to form a large composite dataset	S
MuCo-3DHP [192]	2018	—	Datasets were synthesized from MPI-INF-3DHP by data augmentation	M
3DPW [193]	2018	>50,000	Collect 3D human poses in the field with IMUs and a moving camera	M
		60		
MuPoTS-3D [192]	2018	8,000	Test set of 3D posture estimation for multiple people in the wild	M
		20		
AMASS [194]	2019	N/A	Fifteen different marker-based MoCap datasets were unified into 3D human meshes	S
		(>40 h)		
MoVi [195]	2020	N/A	Large single-player video dataset with 3DMoCap annotations Can provides SMPL parameters obtained through MoSh++	S
		(17 h)		

**Table 8:** Possible posture recognition challenges

Number	Challenges	Description
1	Datasets problems	Lack of special posture datasets and large outdoor 3D datasets.
2	Poor generalization ability	Poor generalization ability leads to low accuracy of posture recognition.
3	Human body occlusion problem	It includes the occlusion of the human body itself, the occlusion of other objects on the human body, and the occlusion of other human bodies on the human body.
4	The contradiction between model accuracy and computational power and large storage space	The increase in the complexity of neural network models leads to an increase in the number of parameters and the demand for computing resources.
5	Depth ambiguity problem	There may be multiple postures in the 3D space that correspond to the human posture in the 2D image.
